# Elastin is a key factor of tumor development in colorectal cancer

**DOI:** 10.1186/s12885-020-6686-x

**Published:** 2020-03-14

**Authors:** Jinzhi Li, Xiaoyue Xu, Yanyan Jiang, Nicole G. Hansbro, Philip M. Hansbro, Jincheng Xu, Gang Liu

**Affiliations:** 1 School of Nursing, Bengbu Medical College, Bengbu, Anhui China; 2 Faculty of Health, University of Technology Sydney, Ultimo, New South Wales Australia; 3 School of Anatomy, Bengbu Medical College, Bengbu, Anhui China; 4 School of Life Sciences, Faculty of Science, University of Technology Sydney, Ultimo, New South Wales Australia; 5 Centre for Inflammation, Centenary Institute, Camperdown, New South Wales Australia; 6 Priority Research Centre for Health Lungs, Hunter Medical Research Institute, The University of Newcastle, New Lambton Heights, New South Wales Australia; 7 Stomatology Department, The First Affiliated Hospital of Bengbu Medical College, Bengbu, Anhui China; 8 School of Dental Medicine, Bengbu Medical College, Bengbu, Anhui China

**Keywords:** Colorectal cancer, Extracellular matrix protein, Elastin, epithelial-mesenchymal transition

## Abstract

**Background:**

Colorectal cancer (CRC) is the most common cancer and a leading cause of death worldwide. Extracellular matrix (ECM) proteins regulate tumor growth and development in CRC. Elastin (ELN) is a component of ECM proteins involved in the tumor microenvironment. However, the role of ELN in CRC remains unclear.

**Methods:**

In this study, we analyzed *ELN* gene expression in tumors from CRC patients and adjacent non-tumor colon tissues and healthy controls from two existing microarray datasets. ELN protein was measured in human normal colon cells and colon cancer epithelial cells and tumor development was assessed in colon epithelial cells cultured in medium with or without ELN peptide on plates coated with ELN recombinant protein. Control plates were coated with PBS only.

**Results:**

We found *ELN* gene expression was increased in tumors from CRC patients compared to adjacent non-tumor tissues and healthy controls. ELN protein was increased in cancer cells compared to normal colon epithelial cells. Transforming growth factor beta (TGF-β) was a key cytokine to induce production of ECM proteins, but it did not induce *ELN* expression in colon cancer cells. Matrix metalloproteinase 9 (*MMP9*) gene expression was increased, but that of *MMP12* (elastase) did not change between CRC patients and control. Tissue inhibitor of metalloproteinases 3 (*TIMP3*) gene expression was decreased in colon tissues from CRC patients compared to healthy controls. However, MMP9, MMP12 and TIMP3 proteins were increased in colon cancer cells. ELN recombinant protein increased proliferation and wound healing in colon cancer epithelial cells. This had further increased in cancer cells incubated in plates coated with recombinant ELN coated plate and in culture media containing ELN peptide. A potential mechanism was that ELN induced epithelial mesenchymal transition with increased alpha-smooth muscle actin and vimentin proteins but decreased E-cadherin protein. Tumor necrosis factor alpha (*TNF*) mRNA was also increased in CRC patients compared to controls. ELN recombinant protein induced further increases in TNF protein in mouse bone marrow derived macrophages after lipopolysaccharide stimulation.

**Conclusions:**

These data suggest ELN regulates tumor development and the microenvironment in CRC.

## Background

Colon cancer, also known as colorectal cancer (CRC), is the third most common cancer diagnosed in both men and women, and it is the second cause of cancer mortality with more than 700,000 deaths worldwide [1]. The exact cause of CRC remains unknown, however polyps have been identified as major precursor to lesions of CRC given approximately 95% of CRC patients have these polyps in their colons [2]. Some other risk factors have also contributed to the prevalence of CRC, including age, cigarette smoke, heavy alcohol consumption, lack of physical activity and genetic family history [3]. Current treatments for CRC aim to block cancer cell growth and metastasis, but there are limited effects on destruction of cancer cells due to the tumor microenvironment in the colon. Therefore, surgery is still the most common therapeutic approach for CRC patients.

Extracellular matrix (ECM) proteins play an important role in regulating cancer cell behaviour and the microenvironment whereby abnormal deposition of ECM results in tissue remodelling and cancer tumorigenesis [4]. ECM proteins are non-cellular structural macromolecules which provide dynamic structural support for integrity and elasticity in all tissues. However, dysregulation of ECM proteins in cancer is characterized by altered tissue structure and composition. Tumors are commonly associated with stiffening structure that mainly induced by high ECM deposition compared to the surrounding tissue [5], however some tumor cells must destroy the ECM structure in order to invade other tissues and metastasise during the latter stages of cancer development [6]. Matrix metalloproteinases (MMPs) are the main enzyme to cleave ECM products, while MMPs associated with their inhibition, tissue inhibitor of metalloproteinases (TIMPs), maintain the level of ECM proteins in tissues [6].

Fibrous protein and proteoglycans are the two main types of ECM proteins [5]. Collagen and elastin (ELN) are fibrous proteins that provide structural integrity and function of tissues. Collagen, a main component of ECM protein provides resistance and support to tissues, while ELN provides the characteristic elasticity properties in many soft tissues [7]. Abnormal level of ELN have been observed in many fibrotic diseases, including kidney [8], lung [9] and liver fibrosis [10]. Studies have shown that fibrosis is a contributing factor in cancer development, and is involved in early stages of CRC [11]. The level of ELN in different cancers has been identified, and an accumulation of ELN fibre is associated with the development of hepatocellular carcinoma [12]. Increased degradation of ELN is found in human breast cancer due to increased activity of MMPs [7]. However, the level of ELN in CRC has not been measured and the role of this molecule in CRC is not well understood.

In this study, we hypothesize that ELN is a key ECM protein regulating tumor growth and development in CRC. We aimed to measure *ELN* gene expression and its related *MMP9, MMP12* and *TIMP3* gene levels in tumor from CRC patients compared to adjacent non-tumor tissues and healthy controls in existing array datasets. We also validated the increased levels of MMPs and TIMP3 proteins in colon cancer cells compared to normal cells. Culture on plates coated with recombinant ELN peptide and in media containing ELN peptide further increases proliferation of colon cancer epithelial cells and induces epithelial mesenchymal transition (EMT). Lipopolysaccharide (LPS)-induced tumor necrosis factor (TNF) secretion by bone marrow derived macrophages (BMDM) are increased after incubation with ELN recombinant protein. Therefore, these data implicate that ELN is a key protein in the tumor microenvironment in CRC and targeting this molecule may help understand the pathogenesis of this disease.

## Methods

### Gene expression in human CRC microarray dataset

Type I alpha I collagen (*COL1A1*), type III alpha I collagen (*COL3A1*), *ELN*, *MMP9*, *MMP12*, *TIMP3*, *TNF* gene expressions were from existing microarray datasets through Gene Expression Omnibus (GEO). The data were analyzed using Bioconductor in R as previously described [13, 14].

In GSE128449 dataset, gene microarray from colorectal tissues were obtained from healthy controls (*n* = 5) and CRC patients (*n* = 31) as shown in Additional file 1: Table S1. Data was profiled by Agilent-014850 Whole Human Genome Microarray 4x44K G4112F.

In GSE110224 dataset, 17 patients with no significant age difference had been histologically confirmed with CRC [15], and tissue specimens of tumor and adjacent non-tumor tissues were collected during surgery. The isolated RNA was previously profiled by Affymetrix Human Genome U133 Plus 2.0 array.

In GSE79462, colon tissues were obtained from resection of CRC patients and healthy controls, and colon organoids were isolated as described previously [16, 17]. The colon organoid cultures were treated with 5 ng/ml recombinant human TGF-β1 protein for 5 days. Total RNA was extracted from cells and microarray data was profiled by Affymetrix Human Genome U133 Plus 2.0 array as previously described [17].

The Benjamini-Hochberg method for adjusted *P* value/false discovery rate (FDR) was used to analyze differences between groups. Statistical significance was set at FDR < 0.05. All target gene expression was calculated as log_2_ intensity robust multi-array average signals (Log_2_ transformed intensity value) [18, 19].

### Cell culture

Normal human colon epithelial cells (FHC, CRL-1831, ATCC, Manassas, VA, USA) were cultured with Dulbecco’s Modified Eagle Medium: Nutrient Mixture F-12 (DMEM/F12) supplemented with 10 mM HEPES, 10 ng/ml cholera toxin, 5 μg/ml insulin, 5 μg/ml transferrin, 100 ng/ml hydrocortisone, 20 ng/ml recombinant epidermal growth factor protein and 10% fetal bovine serum (FBS) at 37 °C with 5% CO_2_.

Human recombinant ELN (10 μg/ml, E6902, Sigma-Aldrich, St. Louis, USA) dissolved in sterile PBS was used to coat cell culture plates overnight at room temperature (RT). Control plates were coated with PBS only [7]. Human colon cancer epithelial cells (Caco-2, HTB-37, ATCC, Manassas, VA, USA) were seeded into each well of 24- or 96-well plate and cultured in Eagle’s Minimum Essential Medium (EMEM) containing 2.5 mM L-glutamine, 10 mM HEPES and 10% FBS at 37 °C with 5% CO_2_. Some cells were cultured in EMEM media with (or without) soluble human ELN peptide (10 μg/ml, ab101300, Abcam, UK). All the cell lines have been authenticated by PCR with short tandem repeat markers, and mycoplasma contamination were checked by using cell culture contamination detection kit (C7028, Thermo Fisher Scientific, MA, USA). The cells were then harvested for cell proliferation and migration assays. mRNA was obtained for qPCR and proteins were collected from cell lysates for immunoblot.

### Cell proliferation assay

Caco-2 cells (1 × 10^5^ cells/well) were grown on 96-well plates coated with (or without) human recombinant ELN as well as treated with culture media with (or without) human ELN peptide. Cell proliferation assays were performed using MTT [3-(4,5-dimethylthiazol-2-yl)-2,5-diphenyltetrazolium bromide] (Sigma-Aldrich, Shanghai, China). Briefly, cell media was changed to serum-free EMDM media after centrifugation (1000 xg, 4 °C for 5 min). MTT was added to each well and incubated for 3 h at RT. MTT solvent was then added to cells and incubated for 15 min with shaking. Optical density (OD) was measured at a wavelength of 590 nm. A standard curve was generated using OD values verses cell number based on the known number of cell populations as previously described [20]. The population of Caco-2 cells was calculated after 6, 12, 24, and 48 h culture in media with (or without) ELN peptide on plates coated with (or without) ELN recombinant protein.

### In vitro wound healing and migration assay

Cacao-2 cells (5 × 10^5^ cells/well) were cultured on a 24-well plate, and starved in serum free EMEM media for 16 h to minimize cell proliferation [21]. A straight line across the centre of each well was scratched using a p200 pipette tip. The cells were then gently washed by three times in PBS to remove detached cells. Fresh serum free EMEM media with (or without) ELN peptide was added and the cells were incubated for a further 48 h at 37 °C with 5% CO_2_. Images were taken using a phase-contrast microscope at 0, 6 and 12-h. The area of each scratch at each time point was compared to the images from 0 h using ImageJ software as previously described [22].

### Protein extraction

Total proteins were extracted from cell lysate using radio immunoprecipitation assay buffer (RIPA; Sigma-Aldrich, St. Louis, USA), supplemented with protease and phosphatase inhibitors (Thermo Fisher Scientific, MA, USA). Lysed cell samples were centrifuged (8000 x*g*, 10 min, 4 °C) and supernatants were collected for protein assays. Total protein concentrations from cell lysates were calculated based on a standard curve from known concentration of albumin using a Pierce bicinchoninic acid (BCA) protein assay kit (Thermo Fisher Scientific, MA, USA) according to the manufacturer’s instructions.

### Immunoblot

Proteins from cell lysates were separated by electrophoresis and transferred onto polyvinylidene difluoride (PVDF) membranes. Membranes were blocked with 5% skim milk for 2 h at RT, and then incubated with anti-elastin (1: 2000, ab23747, Abcam, Cambridge, UK), anti-SMA (1:750, ab5694, Abcam, Cambridge, UK), anti-vimentin (1:2000, ab92547, Abcam, Cambridge, UK), E-cadherin (1: 2000, 3195, Cell signalling technology, USA) and anti-β-actin (1:10,000, ab8226, Abcam, Cambridge, UK) at 4 °C overnight. Blots were incubated with anti-rabbit or anti-mouse IgG HRP conjugated antibodies (R&D System, MN, USA) at RT for 2 h after TBS-Tween-20 wash (3 times, 10 min). Substrate (SuperSignal™ West Femto Maximum sensitivity substrate, Thermo Fisher Scientific, MA, USA) was added to the membrane and images of immunoblots were captured using a ChemiDoc MP System (Bio-Rad, Hercules, USA). Some blots were cut based on molecular weight and some blots were stripped only once for housekeeping protein. Densitometry analysis was performed relative to the housekeeping protein β-actin using ImageJ (NIH, Bethesda, USA) as previously described [23, 24].

### Animals

Six female wild type C57Bl/6 mice aged 5–7 weeks old were obtained from Animal Experimental Center of Beijing Vital River Lab Animal Technology Co. Ltd. (Beijing, China) and maintained at pathogen-free facility in Experiment Center of Bengbu Medical College. Mice were sacrificed at 10 weeks of age by cervical dislocation for bone marrow derived macrophage (BMDM) collection.

### BMDM

L929 cells were seeded into a 75-cm2 flask with 50 ml DMEM media containing 10% FCS 1% L-glutamine and cultured at 37 °C with 5% CO_2_ for 7 days. Supernatant was collected and filtered (0.45 um) as L292-condition media. Bone marrow was isolated from the hind legs of mice by removing connective muscle tissue. The femur and tibia were separated by cutting the joint and bone marrow cells were collected by flushing the bone with 1 ml DMEM media twice (using a 3 ml syringe and 25-gauge needle). The cells were then centrifuged (300 xg, 5 min), resuspended in L292-conditioned media and cultured at 37 °C with 5% CO_2_. Bone marrow cells began to differentiate into BMDMs attached to the flask after 7 days incubation as previously described [25].

### Flow cytometry

Single cell suspensions were obtained from BMDM cell culture. Cells were incubated with Fc block (BD Pharmingen, Franklin Lakes, USA) at 4 °C for 30 min and stained with F4/80 (Conjugated with FITC, BD Pharmingen, Franklin Lakes, USA) antibody at 4 °C for 1 h in the dark. Cells were then enumerated by flow cytometric analysis using a BD LSRFortessa™ flow cytometer with FACSDiva software (BD Biosciences, Franklin Lakes, USA). An anti-mouse Ig, k/Negative compBeads (552,843, BD bioscience, US) was used for single color control and negative control. BMDMs were stained with single color and unstained cells were also used as controls for analysis. Data was analysed using FlowJo software (BD Biosciences, Franklin Lakes, USA) as previously described [26, 27].

### Enzyme-linked immunosorbent assay (ELISA)

The concentration of TNF in mouse BMDMs was determined using DuoSet ELISA kits (DY410, R&D systems, Minneapolis, USA) according to the manufacturer’s instructions. The standard curve in this kit ranges from 31.3 to 2000 pg/ml. Each sample, including standards are triplicated and only coefficient of variation less than 6 were used in this study. The concentration of TNF in BMDMs was normalized to the total protein concentration.

### Statistical analysis

Results are presented as mean ± standard error of the mean (SEM). Each in vitro experiment was performed in triplicate and repeated in three or four independent experiments. Unpaired student *t*-Tests were used to compare two groups in existing dataset analysis and cell culture experiments. A one-way analysis of variance (ANOVA) with Bonferroni comparisons was used to compare between multiple groups. All statistical analyses were performed using GraphPad Prism Software (San Diego, CA, USA).

## Results

### Collagen and ELN are increased in patients with CRC

Collagen is the most abundant ECM protein and previous studies have shown a correlation of increased collagen expression with CRC [28]. To confirm this finding, we assessed *COL1A1* expression, the most abundant molecule of the collagen family, in tumor tissues from CRC patients and colon tissues from healthy controls using an existing dataset (GSE128449). *COL1A1* expression was significantly increased in tumors from CRC patients compared to colon tissues from healthy controls (*P* < 0.003, Additional file 1: Fig. S1A). *COL3A1* is another important component in the collagen family, and *COL3A1* expression was also significantly increased in CRC patients compared to healthy controls (*P* < 0.03, Additional file 1: Fig. S1B). To examine whether the increased collagen was associated with tumors development, we next assessed the level of collagen mRNA expression in tumors and adjacent non-tumor colon tissues from the same CRC patients (GSE110224). *COL1A1* and *COL3A1* mRNA expression was significantly increased in tumor tissue compared to adjacent non-tumor tissues in colons from CRC patients (*P* < 0.0001 and *P* < 0.03, Additional file 1: Fig. S1C and D).

**Fig. 1:**
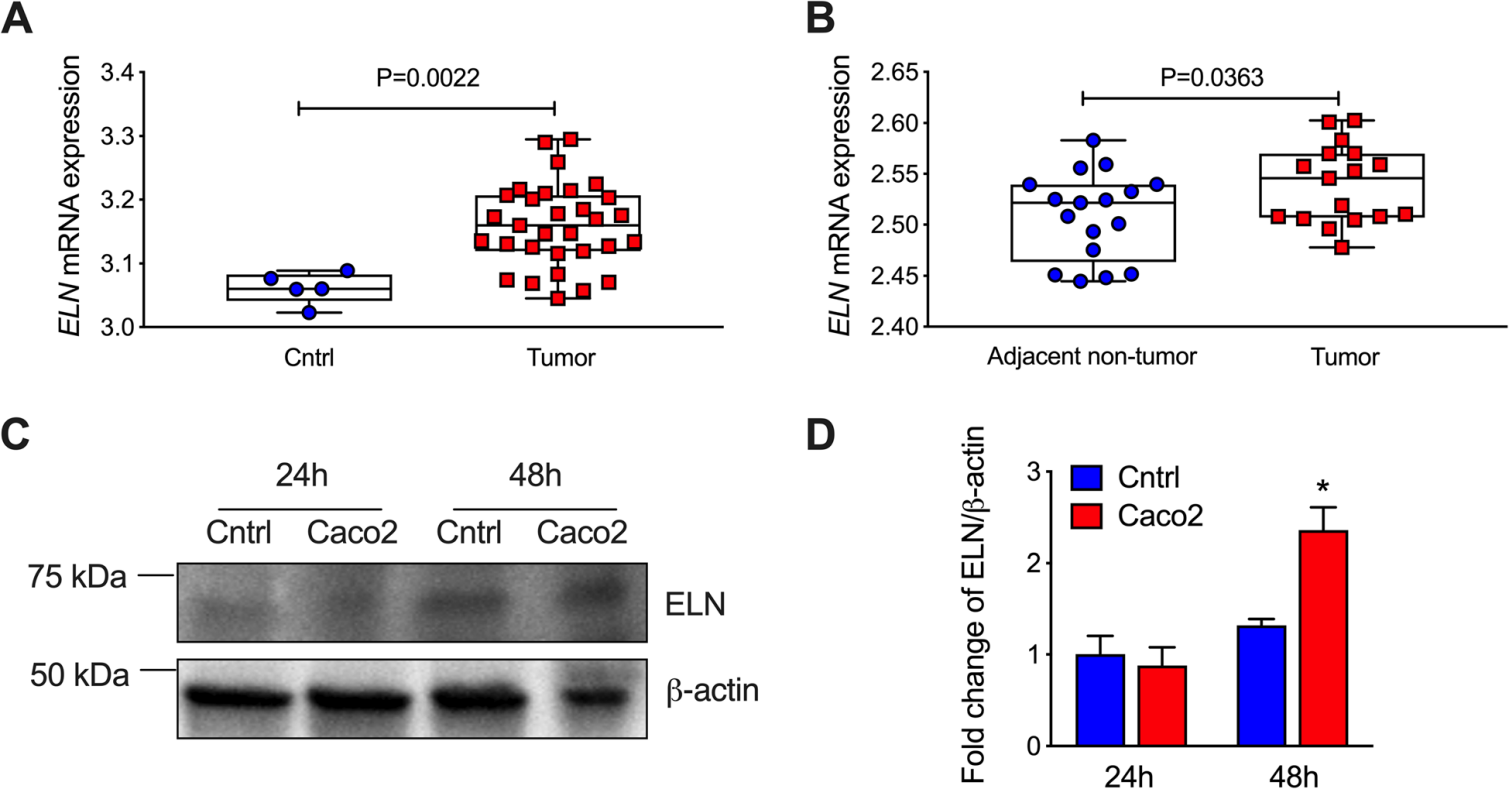
*ELN* mRNA expression is increased in colorectal cancer (CRC) patients, and ELN protein is increased in colon cancer epithelial cells. **a***ELN* gene expression was analyzed from colon tissues from healthy controls (*n* = 5) and CRC patients (*n* = 31) based on a GSE128449 dataset. **b***ELN* gene expression was analyzed from colon tissues from tumor and adjacent non-tumor tissues from the same CRC patients (*n* = 17) according to a GSE110224 dataset. Colon cancer epithelial cells and normal colon epithelial cells were cultured. **c** ELN protein was assessed in cell lysates after 24 and 48 h incubation by immunoblot, and **d** fold change of densitometry normalized to β-actin, *n* = 4. Results are mean ± SEM. *P < 0.05 compared to normal colon epithelial cells

We then assessed the level of *ELN* in CRC patients using the same microarray datasets. We found that *ELN* mRNA expression was significantly increased in tumor tissues from CRC patients compared to those from healthy controls (*P* = 0.0022, Fig. 1a). There was also significantly increased *ELN* mRNA expression in tumors from CRC patients compared to adjacent non-tumor tissues (*P* = 0.0363, Fig. 1b). We next assessed the level of ELN protein in human colon cancer epithelial cells compare to normal colon epithelial cells (Fig. 1c, Additional file 1: Fig. S2). ELN protein was significantly increased in cancer cells after 48 h incubation compared to normal colon cells, although ELN protein was not changed at the early time point (Fig. 1d). These results indicate that increased ELN is associated with tumor development in CRC patients.

### TGF-β does not affect *ELN*, but *MMP9* and *TIMP3* are associated with increased *ELN* mRNA in CRC development

**Fig. 2:**
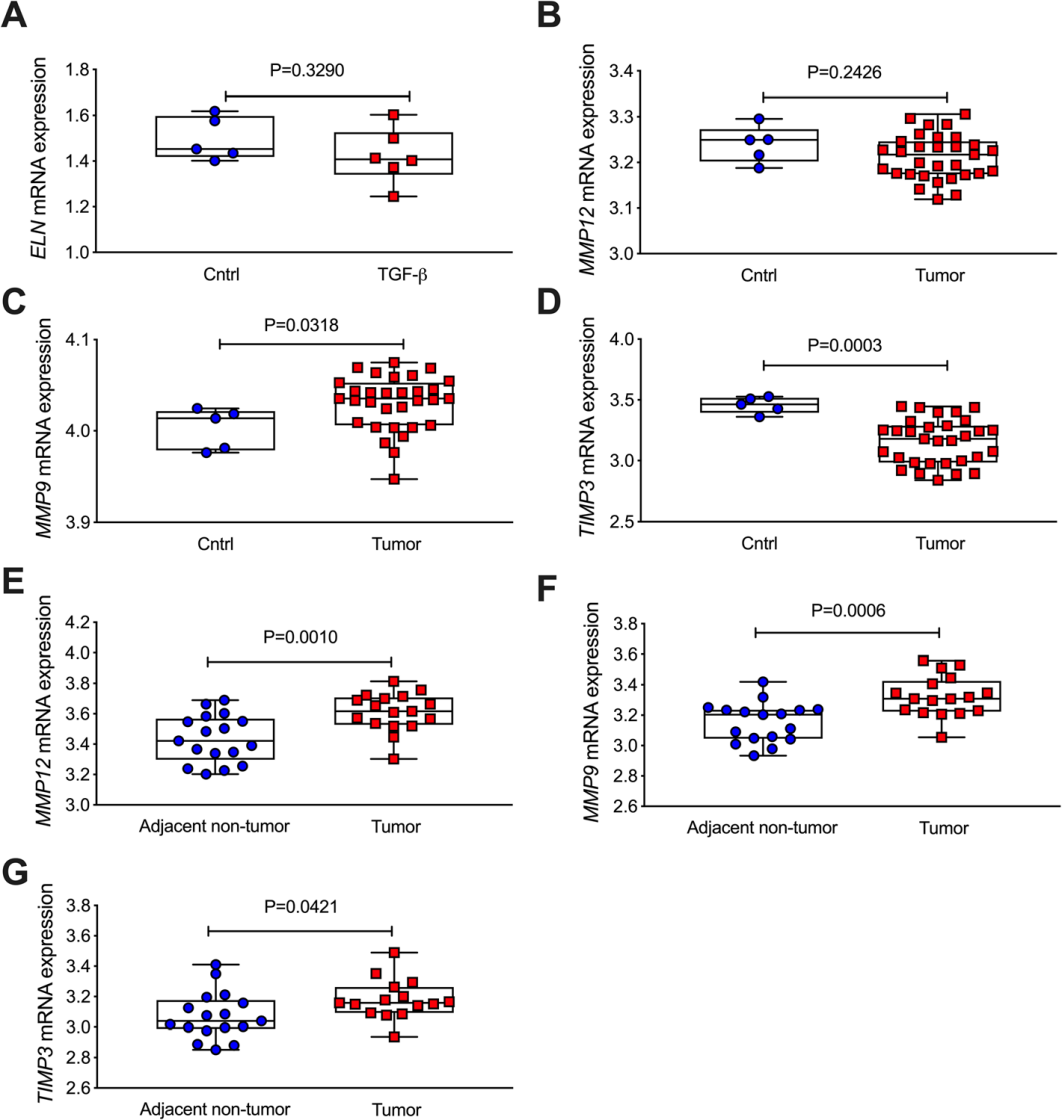
*ELN* mRNA is not changed, but abnormal levels of *MMP12*, *MMP9* and *TIMP3* mRNA expression are found in tumor from CRC patients compared to adjacent non-tumor colon tissues and healthy controls. **a** Colon organoid were isolated from tumor tissues in CRC patients and healthy controls. The organoids were stimulated with TGF-β for 5 days, and *ELN* mRNA expression was measured from microarray data based on a GSE79462 dataset (*n* = 5–6). *MMP12* (**b**), *MMP9* (**c**) and *TIMP3* (**d**) gene expression was analyzed from colon tissues from healthy controls (*n* = 5) and CRC patients (*n* = 31) based on a GSE128449 dataset. *MMP12* (**e**), *MMP9* (**f**) and *TIMP3* (**g**) gene expression was analyzed from colon tissues from tumor and adjacent non-tumor tissues from the same CRC patients (*n* = 17) according to a GSE110224 dataset. Results are mean ± SEM

**Fig. 3:**
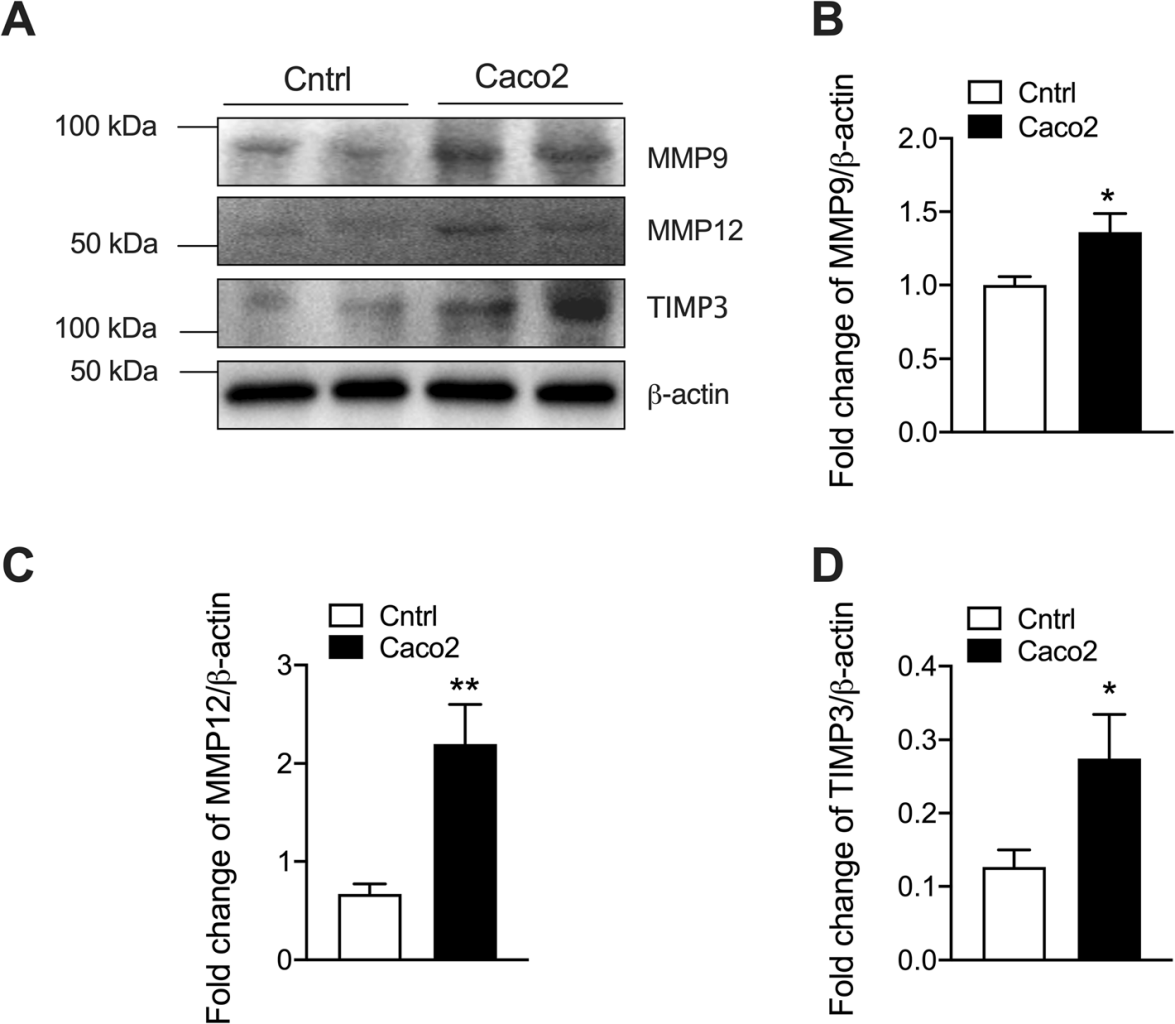
MMP9, MMP12 and TIMP3 proteins are increased in colon cancer epithelial cells compared to normal colon cells. Colon cancer epithelial cells (Caco-2) and normal colon epithelial cells were cultured. **a** MMP12, MMP9 and TIMP3 protein was assessed in cell lysates after 48 h incubation by immunoblot. Fold change of densitometry of MMP12 (**b**), MMP9 (**c**) and TIMP3 (**d**) normalized to β-actin, *n* = 4. Results are mean ± SEM. *P < 0.05, **P < 0.01 compared to normal colon epithelial cells. Uncropped blots were shown in Additional file 1: Fig. S3

Previous studies have shown that TGF-β is a main growth factor to induce the productions of ECM proteins in cells [29]. Given increased *ELN* was found in CRC patients, we then determined whether TGF-β induced *ELN* expression in colon organoids from CRC patients and healthy controls according to an existing dataset (GSE79462) [17]. TGF-β challenge did not affect *ELN* mRNA level in colon organoids from CRC patients or controls (*P* = 0.329, Fig. 2a). To further explore the mechanism of increased ELN in CRC, we then assessed the level of MMP expression in colon tissues from CRC patients and healthy controls. MMP12 is the main enzyme to catalyse ELN, and we found that *MMP12* expression was not changed between tumors from CRC patients and controls (*P* = 0.2426, Fig. 2b). However, *MMP9* gene expression was increased in tumors from CRC patients compared to colons in healthy controls (*P* = 0.0318, Fig. 2c). TIMP3 is an inhibitor of MMPs and its gene expression was significantly decreased in tumor tissues compared to controls (*P* = 0.0003, Fig. 2d). We then assessed *MMPs and TIMP3* expression in tumors and adjacent non-tumor colon tissue in CRC patients, *MMP12* (*P* = 0.001, Fig. 2e), *MMP9* (*P* = 0.0006, Fig. 3f) and *TIMP3* (*P* = 0.0421, Fig. 2g) mRNA expression was significantly increased in tumors tissue compared to adjacent non-tumor tissue from CRC patients.

### MMP9, MMP12 and TIMP3 protein is increased in colon cancer cells

Given abnormal levels of *MMP9*, *MMP12* and *TIMP3* mRNA expression in tumors from CRC patients and controls, we next measured the level of the MMPs and TIMP3 proteins in human colon cancer epithelial cells compared to normal colon cells by immunoblot (Fig. 3a, Additional file 1: Fig. S3). ELN proteins had the high level in cancer cells after 48 h culture compared to normal colon epithelial cells (Fig. 1d). Thus, we chose this time point to measure the protein level of MMPs. Both MMP9 and MMP12 proteins were increased in colon cancer epithelial cells compared to controls (Fig. 3b and c), with MMP12 proteins having more than 2-fold increase, while MMP9 proteins had a 1.3-fold increase in cancer cells compared to normal cells. TIMP3 protein was also measured in cancer and normal colon epithelial cells. Colon cancer epithelial cells had higher levels of TIMP3 protein than normal colon epithelial cells (Fig. 3d).

### ELN induces colon epithelial cancer cell migration and proliferation

**Fig. 4:**
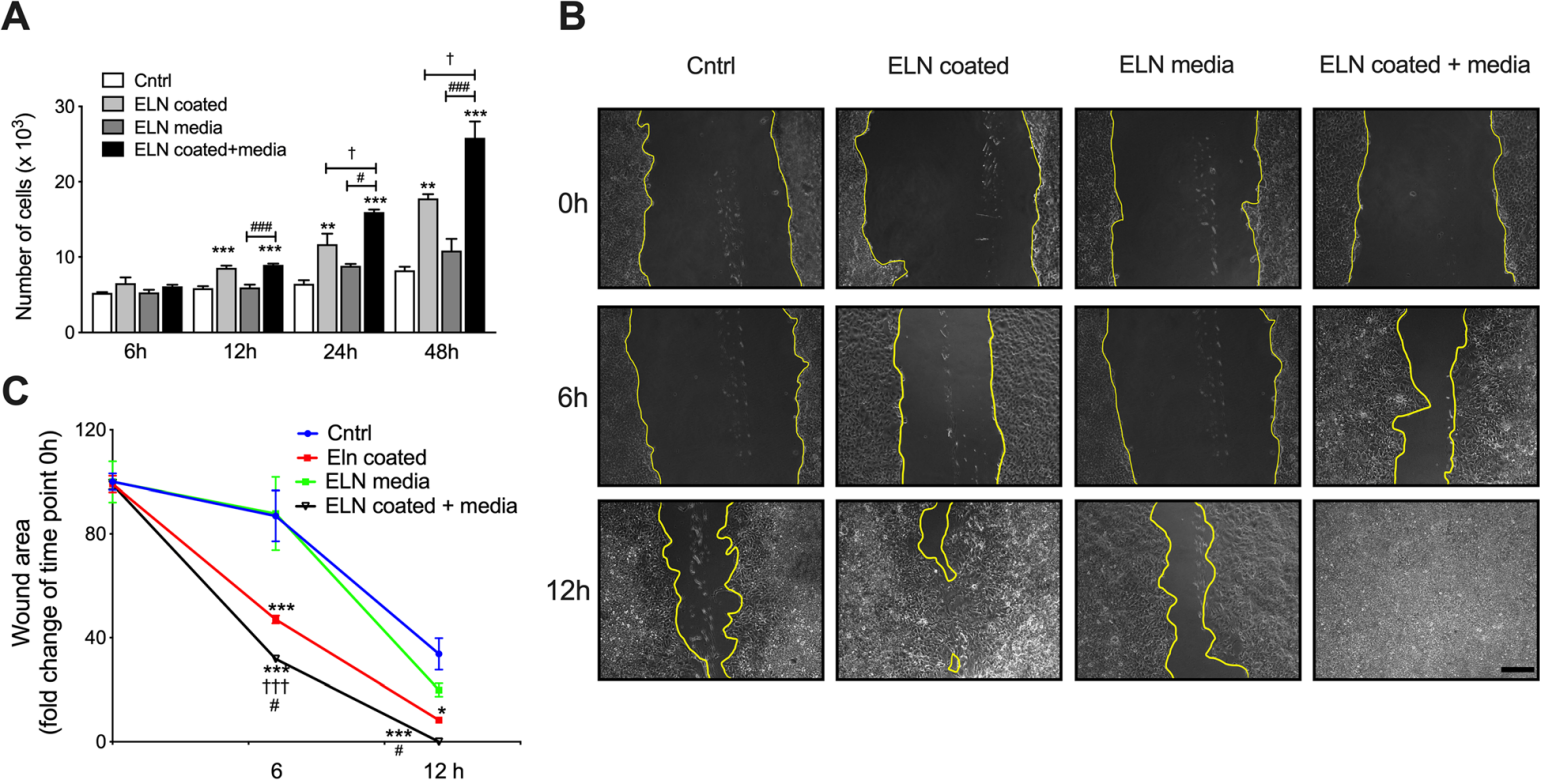
ELN increases human colon cancer epithelial cell proliferation and invasion. Human colon cancer epithelial cells (Caco-2) were seeded onto plates coated with recombinant ELN protein and cultured in medium with (or without) ELN peptide. Control plates were coated with PBS only. **a** Cell proliferation was assessed after 6, 12, 24 and 48 h incubation by MTT assay. **b** A straight scratch was performed in each well and cancer cells invasion and migration were assessed by wound healing. **c** Wound area was assessed by measuring the wound closure size after 6 and 12 h post scratch, scale bar is 200 μm. *n* = 4. Results are mean ± SEM. *P < 0.05, ***P < 0.001 compared to cells cultured in normal medium on control plates. ††† < 0.001, compared to cells cultured in medium contain ELN peptide on control plates. #*P* < 0.05 compared to cells cultured in normal medium on ELN coated plates

To investigate the role ELN plays in regulating tumor development in CRC, we seeded human colon cancer epithelial cells onto culture plates coated with (or without) recombinant ELN protein for 48 h. Cell proliferation was measured by MTT after 6, 12, 24, and 48 h. Cancer cell number were not changed after 6 h culture, but significantly increased after 12 h incubation with ELN recombinant protein compared to controls (Fig. 4a). There was a further increase in cell proliferation after 24 and 48 h incubation with ELN recombinant protein compared to controls. To further explore the role of ELN in colon tumor growth, we seeded human colon cancer epithelial cells onto plates coated with ELN recombinant protein and incubated in medium with (or without) ELN peptide. Cell numbers further increased in plates coated with ELN and media containing ELN peptide compared to cells cultured on plates coated with ELN alone. We next determined whether ELN regulates cancer cell migration by performing a wound healing assay (Fig. 4b). ELN recombinant protein increased cancer cell growth and reduced wound area after 6 h incubation compared to PBS controls. Colon cancer cell migration had further increased after 6 h incubation onto plates coated with recombinant ELN as well as in media containing ELN peptide. The wound area had recovered after 12 h incubation in plates coated with ELN recombinant protein in plates coated with ELN and media containing ELN peptide (Fig. 4c). This indicates ELN induces human colon epithelial cell growth and migration.

### ELN induced epithelial-mesenchymal transition (EMT) in human epithelial cancer cells

**Fig. 5:**
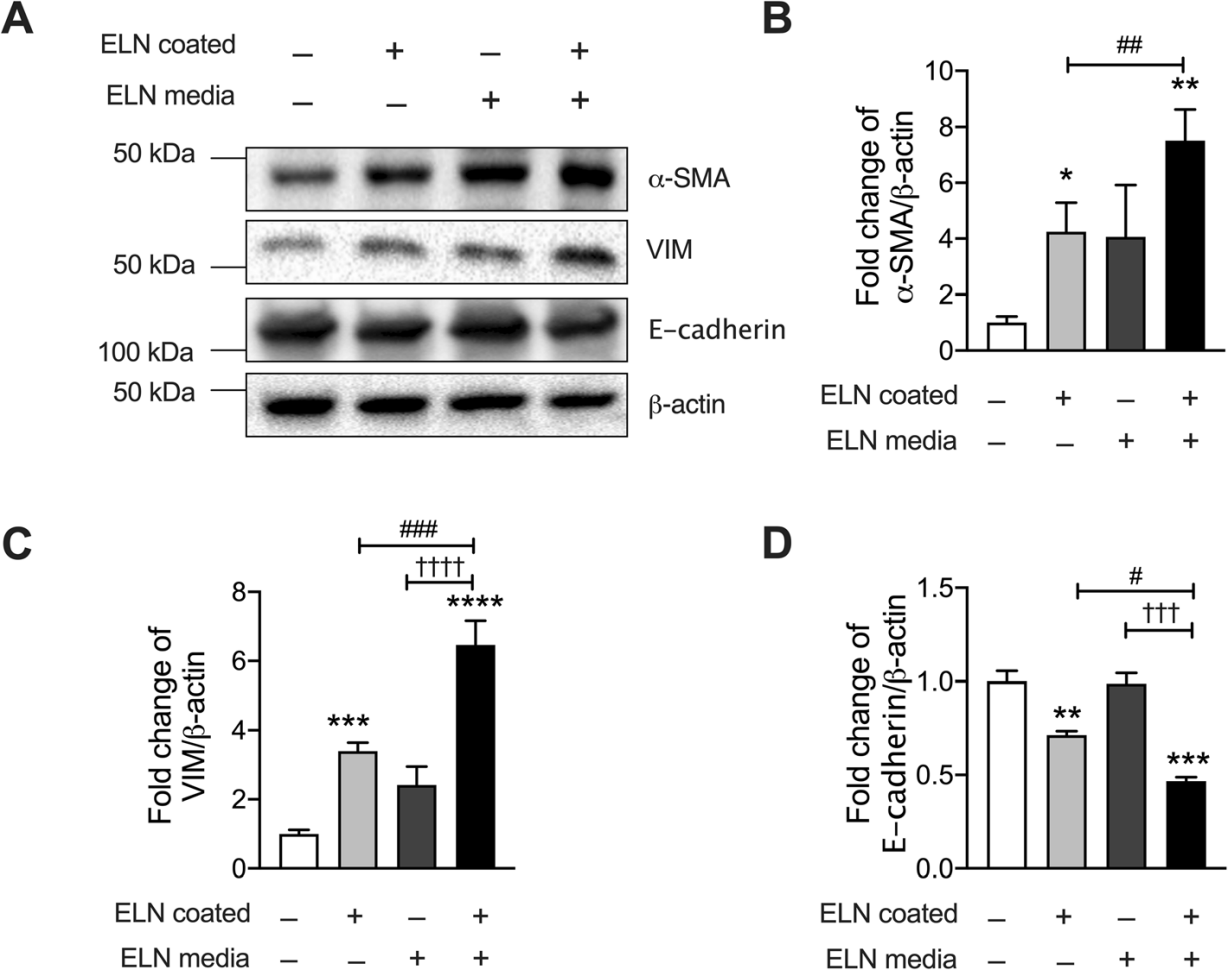
ELN induces epithelial mesenchymal transition (EMT) in human colon cancer epithelial cells. Human colon cancer epithelial cells (Caco-2) were seeded onto plates coated with recombinant elastin proteins. Control plates were coated with PBS only. Cells were cultured in medium with (or without) ELN peptide for 48 h **a** Total protein were collected from cell lysates and α-SMA, vimentin (VIM) and E-cadherin proteins were assessed by immunoblot. Fold change of densitometry of **b** α-SMA **c** VIM and **d** E-cadherin were normalized to β-actin. *n* = 4, Results are mean ± SEM. *P < 0.05, **P < 0.01, ***P < 0.001, ****P < 0.0001 compared to cells cultured in normal media on control plates. ††† < 0.001, †††† < 0.0001 compared to cells cultured in medium containing ELN peptide on control plates. #*P* < 0.05, ##*P* < 0.01, ###*P* < 0.001 compared to cell cultured in normal medium on ELN coated plates. Uncropped blots were shown in Additional file 1: Fig. S4

EMT is a key process to induce cancer epithelial cell migration and invasion [30]. To further investigate the role of ELN in the regulation of tumor development in CRC, we measured EMT markers alpha smooth muscle actin (α-SMA) and vimentin (VIM) [31] in human colon cancer epithelial cells cultured on plates collated with (or without) ELN recombinant protein and with (or without) media containing ELN peptide for 48 h by immunoblot (Fig. 5a, Additional file 1: Fig. S4). The level of α-SMA protein in colon cancer epithelial cells had increased coated plate compared to controls (Fig. 5b). In addition, the level of VIM protein was also significantly increased in cancer cells after incubation with ELN protein and peptide compared to controls (Fig. 5c), indicating elastin proteins induced EMT in colon epithelial cancer cells. E-cadherin (a marker of epithelial cells) was also measured in colon cancer epithelial cells incubated on plates coated with (or without) recombinant ELN and with (or without) media containing ELN peptide for 48 h. E-cadherin proteins were significantly decreased in colon cancer epithelial cells after 48 h on plates coated with recombinant ELN alone. E-cadherin proteins had a further decreased in cells following culture on plates coated with recombinant ELN as well as in media containing ELN peptide.

### *TNF* gene expression is increased in CRC patients and ELN induces bone marrow derived macrophages (BMDM) to secrete TNF

**Fig. 6:**
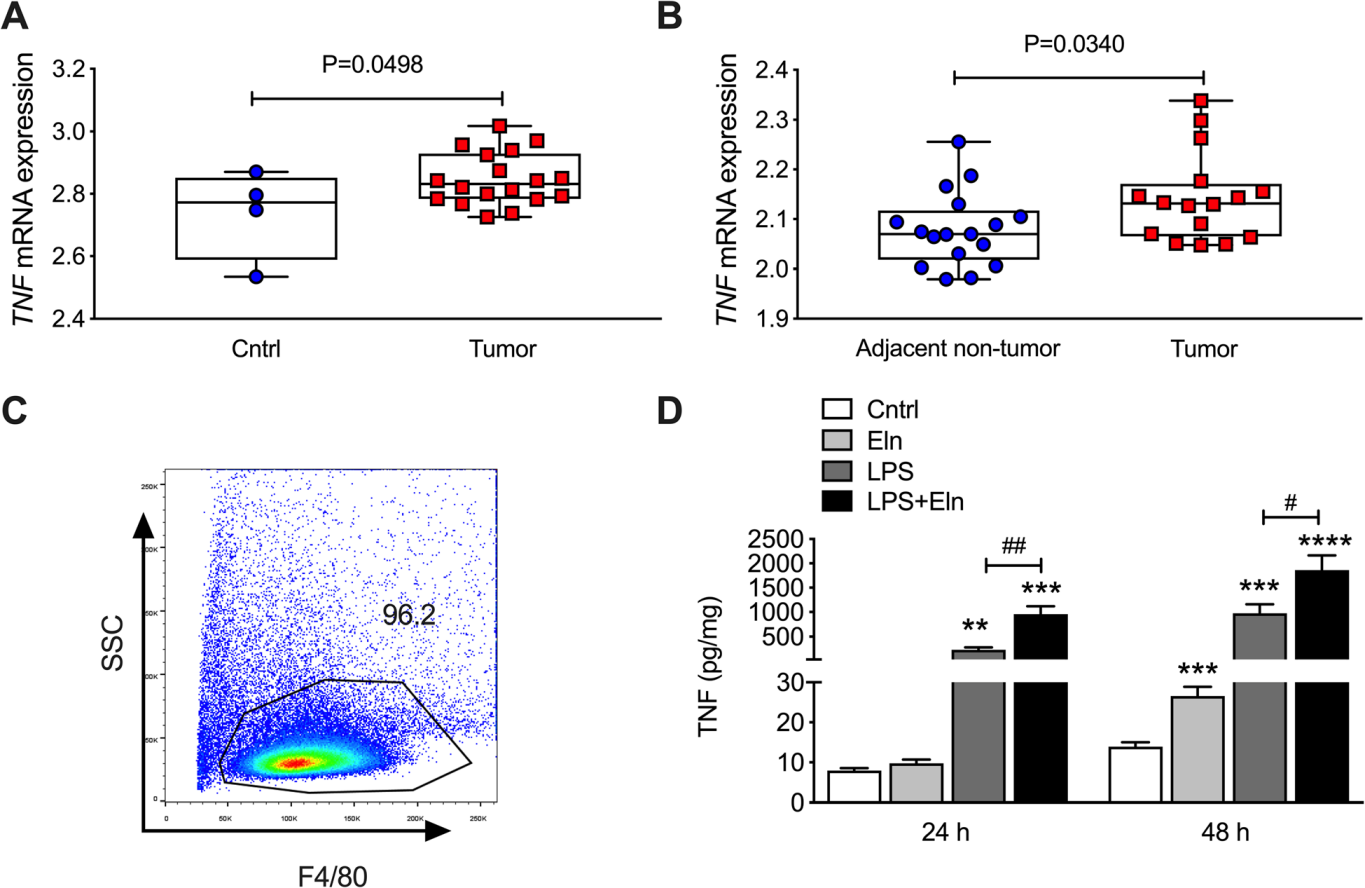
*TNF* mRNA is increased in CRC patients and ELN recombinant protein increases TNF protein from bone marrow derived macrophages (BMDM) after LPS challenge. **a***TNF* gene expression was analyzed from colon tissues from healthy controls (*n* = 5) and CRC patients (*n* = 31) based on a GSE128449 dataset. **b***TNF* gene expression was analyzed from colon tissues from tumor and adjacent non-tumor tissues from the same CRC patients (*n* = 17) according to a GSE110224 dataset. **c** BMDM were isolated from mice and the purity of macrophages were assessed by flow cytometry. **d** BMDM were cultured on plates coated with recombinant ELN protein, and control plates were coated with PBS only. BMDM cell lysates were collected 24 and 48 h after LPS challenge, and TNF proteins measured by ELISA. *n* = 6. Results are mean ± SEM. **P < 0.01, ***P < 0.001 compared to control plates. #*P* < 0.05 compared to plates coated with LPS only

Inflammation is a key factor in inducing tumor development, and cytokines, such as TNF involved in chronic inflammation contributed to this microenvironment [32]. We found that *TNF* mRNA expression was significantly increased in colon tumor tissues from CRC patients compared to healthy controls (*P* = 0.0498, Fig. 6a). *TNF* mRNA expression was also increased in tumors from CRC patients compared to adjacent non-tumor tissues in colons from the same patients (*P* = 0.034, Fig. 6b). Macrophages are the main cell types secreting TNF and therefore involved in tumor formation in CRC [33]. To further understand the role of ELN in regulating tumor development in CRC, we isolated BMDM from mice with over 96% purity (Fig. 6c). Lipopolysaccharide (LPS) is a gram-negative bacteria antigen involved in inflammation and colorectal cancer progression. BMDMs were cultured with recombinant ELN proteins with (or without) LPS challenge. Cell lysates were collected, and TNF protein was measured after 24 h and 48 h incubation by ELISA. ELN recombinant protein induced TNF protein secretion after 48 h compared to controls (Fig. 6d). However, ELN treated cells had further increased TNF protein secretion after LPS challenge compared to LPS only. This indicates that ELN protein activates macrophages to release TNF thereby changing the microenvironment in local tissues.

## Discussion

ECM proteins are important macromolecules contributing to the microenvironment in tumor development in many cancers, including CRC. ELN is a key component of the ECM family and plays critical roles in elasticity properties in soft tissues and may contribute to tumor formation. Previous studies have shown that ELN promotes human breast cancer cell invasiveness [7], indicating a potential role of ELN in cancer cell migration. However, the level of ELN in CRC has not been measured and the role of this molecule in CRC is not fully understood. We are the first to measure *ELN* gene expression in CRC from two existing microarray datasets. *ELN* mRNA expression is increased in tumors from CRC patients compared to normal colon tissues from healthy controls, and in tumor tissue compared to adjacent non-tumor colon tissues from the same CRC patients. We have also found increased ELN protein in cancer cells compared to normal colon epithelial cells. *MMP12* gene expression is not changed, but *MMP9* mRNA expression is increased in tumor from CRC patients compared to adjacent non-tumor tissues and controls. *TIMP3* gene decreased in CRC patients compared to controls and slightly increased in tumor tissues compared to adjacent none-tumor tissues. The protein levels of MMP12, MMP9 and TIMP3 are increased in cancer cells compared to controls after 48 h culture. In this study, we are the first to show that ELN recombinant protein further induced proliferation and migration of human colon cancer epithelial cells. The ELN recombinant protein also induces α-SMA and VIM proteins, but reduces E-cadherin in colon cancer epithelial cells. Chronic inflammation is also involved in CRC and we show that *TNF* gene expression is increased in CRC patients. LPS challenge increased TNF protein levels in BMDM and this was further increased after incubation with ELN recombinant protein.

Changes to ECM deposition in colon cell has contributed to the tumor microenvironment and ECM stiffening that is associated with tumor progression. Collagen is the most abundant protein in the ECM and increased deposition of collagen contributes to tissue remodelling and tumor formation. Previous studies have shown that collagen proteins are increased in CRC patients [34]. *COL1A1* and *COL3A1* are main two molecules in the collagen family, and increased COL1A1 and COL3A1 ratio induces tissue stiffening [35]. Our results are consistent with previous studies that gene expression of these two molecules is increased in CRC patients, and tumor tissues have more collagen expression than adjacent non-tumor tissues.

Increased *ELN* mRNA expression is found in tumors from CRC patients compared to non-tumor tissue and healthy controls. ELN proteins are also increased in cancer cells compared to normal colon epithelial cells. To understand the mechanism of increased ELN in CRC, we measure *ELN* gene expression in cancer cells after TGF-β stimulation. TGF-β is a fibrotic cytokine that induces ECM proteins in many diseases [24]. However, the level of *ELN* gene expression does not change between cells treated with TGF-β recombinant protein and controls, indicating TGF-β is not involved in the induction of *ELN* expression in CRC. MMPs and TIMPs are the main enzymes involved in maintaining ECM levels, including ELN. *MMP12* gene expression is not changed between tumors from CRC patients and controls. *TIMP3* gene expression decreased in tumor tissue in CRC patients compared to healthy control, but its levels are increased in tumors compared to adjacent non-tumor tissues. However, the protein levels of MMP9, MMP12 and TIMP3 are increased in cancer cells. Dysregulation of MMP gene expression and protein levels may not be able to explain the increased ELN level in tumor tissues from CRC patients. The enzyme activity of MMPs may need to be further explored. The levels and roles of MMPs and TIMPs are diverse during cancer development. There are also many factors affecting MMP expression in the tumor microenvironment, including the types of cancer cells and surrounding stromal cells [36]. In addition, the role of MMPs may switch from stimulation of cancer cell growth to protection at different stages during cancer progression [37, 38]. This may partially explain why the gene and protein levels of MMP9 and MMP12 are different in CRC patients.

ELN was originally identified as an adhesion molecule in many tissues that are involved in cell-to-cell or cell-to-ECM interaction. The role of ELN in tumor development and metastasis has not been fully understood. Previous studies show that ELN rapidly binds to lung carcinoma and melanoma cells, indicating ELN has a high metastatic capacity [39]. In addition, tumor cell invasion is commonly facilitated within ELN rich pulmonary tissue [40]. We have shown here that ELN recombinant protein increases proliferation and invasion of human colon cancer epithelial cells, indicating ELN plays an important role in regulating cancer cell adhesion, migration and invasion. While the mechanisms of ELN induced cancer cell migration remain unclear, EMT is an important process in tumor development and many ECM proteins have been identified to induce EMT. Previous studies have shown that COL1A1 promotes airway epithelial cells to develop a mesenchymal cell phenotype in lung cancer [41]. A potential relationship between ELN and EMT has been identified in organ fibrosis [42] and bronchopulmonary dysplasia [43]. ELN and collagen together may be associated with EMT in non-small cell lung cancer [44]. In this study, we have shown that human colon cancer epithelial cells lose their epithelial marker, but expressed mesenchymal markers after incubation with ELN recombinant protein. Therefore, a potential mechanism is that ELN induces the EMT process in colon tissues and increases tumor cell proliferation and invasion. The use of ELN functional peptide in hydrogel assays may be used to provide a 3D culture system, however the lack of commercial material limits this work. In this study, we cultured cancer cells in media containing ELN peptide on plates coated with ELN recombinant protein to maximize the ELN rich environment in cancer cells.

Chronic inflammation, a main factor of the tumor microenvironment induces cancer risk in colon tissues, and immune cells affect tumor development through cytokines, chemokines and growth factors [32]. Previous studies demonstrate that tumor-associated macrophages express high level of proinflammatory cytokines, such as TNF to promote tumor growth and invasion [45, 46]. We have shown here that ELN recombinant protein induces macrophages to secrete TNF. Infection also elevates the risk of cancer development in CRC, and LPS from gram negative bacteria has been identified to contribute to cancer metastasis in CRC [47]. LPS challenge increased macrophages to secrete TNF products, and ELN recombinant protein further increased TNF after LPS challenge. In addition, inflammation was thought to be a driver of ECM production and remodelling. However, recent studies have shown that inflammation and abnormal deposition of ECM proteins are two independent pathways. We have previously shown that ECM proteins regulate inflammation in chronic diseases [23, 26]. Here, we have demonstrated that ELN induces macrophages involved in inflammation. This provides further evidence that ECM proteins regulate inflammation.

## Conclusions

Taken together, our data show that ELN plays an important role in regulating tumor cell proliferation and invasion in CRC. Identification of ELN responsible for CRC may provide an opportunity to develop a new therapeutic approach and exert a major impact on improving the outcome of CRC patients.

## Supplementary information

**Additional file 1: Table S1.** Human subject characteristics from dataset GSE128449. **Fig. S1.** Type I alpha 1 collagen (COL1A1) and type III alpha 1 collagen (COL3A1) mRNA expression are increased in tumor tissues compared to adjacent non-tumor colon tissues from colorectal cancer (CRC) patients. *COL1A1* (A) and *COL3A1* (B) mRNA expression was assessed from colon tissues from healthy controls (*n* = 5) and CRC patients (*n* = 31) based on a GSE128449 dataset. *COL1A1* (C) and *COL3A1* (D) gene expression was measured from colon tissues from tumor and adjacent non-tumor normal tissues from the same CRC patients (*n* = 17) according to a GSE110224 dataset. Results are mean ± SEM. **Fig. S2.** Full length immunoblots of ELN and β-actin in Fig. 1c. ELN was probed and the same blot was stripped for β-actin detection by immunoblot Molecular ladder was shown on the side of each immunoblot image. Red rectangle indicates the cropped representative image in Fig. 1c. **Fig. S3.** Full length immunoblots of MMP12, MMP9, TIMP3 and β-actin in Fig. 3. Molecular ladder was shown on the side of each immunoblot image. Red rectangle indicates the cropped representative image in Fig. 3. MMP12 blot were cut and incubated with β-actin after stripping. **Fig. S4**. Full length immunoblots of α-SMA, VIM, E-cadherin and β-actin in Fig. 5. Molecular ladder was shown on the side of each immunoblot image. Red rectangle indicates the cropped representative image as shown in Fig. 5.

## Data Availability

The data analyzed during the current study are available from the corresponding author on reasonable request.

## Abbreviations

BMDM: Bone marrow derived macrophages
COL1A1: Type I alpha 1 collagen
COL3A1: Type III alpha 1 collagen
CRC: Colorectal cancer
ECM: Extracellular matrix
ELN: Elastin
EMT: Epithelial mesenchymal transition
FBS: Fetal bovine serum
FDR: False discovery rate
GEO: Gene expression omnibus
LPS: Lipopolysaccharide
MMP: Matrix metalloproteinase
OD: Optical density
TGF-β: Transforming growth factor beta
TIMP: Tissue inhibitor of metalloproteinase
TNF: Tumor necrosis factor
VIM: Vimentin gene
α-SMA: Alpha smooth muscle actin
