# Supplementary material for: Elastin is a key factor of tumor development in colorectal cancer
Source: BMC Cancer. 2020 Mar 14;20:217. doi: 10.1186/s12885-020-6686-x (PMC7071655; doi:10.1186/s12885-020-6686-x)
Supplement: Supplementary file 1 — Additional file 1: Table S1. Human subject characteristics from dataset GSE128449. Fig. S1. Type I alpha 1 collagen (COL1A1) and type III alpha 1 collagen (COL3A1) mRNA expression are increased in tumor tissues compared to adjacent non-tumor colon tissues from colorectal cancer (CRC) patients. COL1A1 (A) and COL3A1 (B) mRNA expression was assessed from colon tissues from healthy controls (n = 5) and CRC patients (n = 31) based on a GSE128449 dataset. COL1A1 (C) and COL3A1 (D) gene expression was measured from colon tissues from tumor and adjacent non-tumor normal tissues from the same CRC patients (n = 17) according to a GSE110224 dataset. Results are mean ± SEM. Fig. S2. Full length immunoblots of ELN and β-actin in Fig. 1c. ELN was probed and the same blot was stripped for β-actin detection by immunoblot Molecular ladder was shown on the side of each immunoblot image. Red rectangle indicates the cropped representative image in Fig. 1c. Fig. S3. Full length immunoblots of MMP12, MMP9, TIMP3 and β-actin in Fig. 3. Molecular ladder was shown on the side of each immunoblot image. Red rectangle indicates the cropped representative image in Fig. 3. MMP12 blot were cut and incubated with β-actin after stripping. Fig. S4. Full length immunoblots of α-SMA, VIM, E-cadherin and β-actin in Fig. 5. Molecular ladder was shown on the side of each immunoblot image. Red rectangle indicates the cropped representative image as shown in Fig. 5. [file 12885_2020_6686_MOESM1_ESM.docx]

# Elastin is a key factor of tumor development in colorectal cancer

Jinzhi Li^1^_†_, Xiaoyue Xu^2,^_†_, Yanyan Jiang^3^, Nicole G. Hansbro^4,5,6^, Philip M Hansbro^4,5,6^, Jincheng Xu^7, 8^ *and Gang Liu^4,5^*

^1^School of Nursing, Bengbu Medical College, Bengbu, Anhui, China

^2^Faculty of Health, University of Technology Sydney, Ultimo, New South Wales, Australia.

^3^School of Anatomy, Bengbu Medical College, Bengbu, Anhui, China

^4^School of Life Science, Faculty of Science, University of Technology Sydney, Ultimo, New South Wales, Australia

^5^Centre for Inflammation, Centenary Institute, Camperdown, New South Wales, Australia

^6^Priority Research Centre for Health Lungs, Hunter Medical Research Institute, The University of Newcastle, New Lambton Heights, New South Wales, Australia

^7^Stomatology Department, The first affiliated hospital of Bengbu Medical College Bengbu, Anhui, China

^8^School of Dental Medicine, Bengbu Medical College, Bengbu, Anhui, China

†These authors contributed equality to this work and should be considered co-first authors.

***Corresponding author:**

Dr Gang Liu, Centre for Inflammation, School of Life Science, University of Technology Sydney, Ultimo, New South Wales, Australia. Email: Gang.Liu@uts.edu.au.

Professor Jincheng Xu, Stomatology Department, The first affiliated hospital of Bengbu Medical College, Bengbu, Anhui, China. Email: xjch9999@163.com

**Table S1.** Human subject characteristics from dataset GSE128449

|  | **Healthy** | **CRC** | **p-value** |
| --- | --- | --- | --- |
| Number | 5 | 31 | N/A |
| Sex (% female) | 60% | 61.20% | P=0.9 |
| Median Age (range) | 65 (64-77) | 73 (39-86) | P=0.5858 |

CRC: colorectal cancer; N/A: non-applicable

**Supporting figures and figure legends**


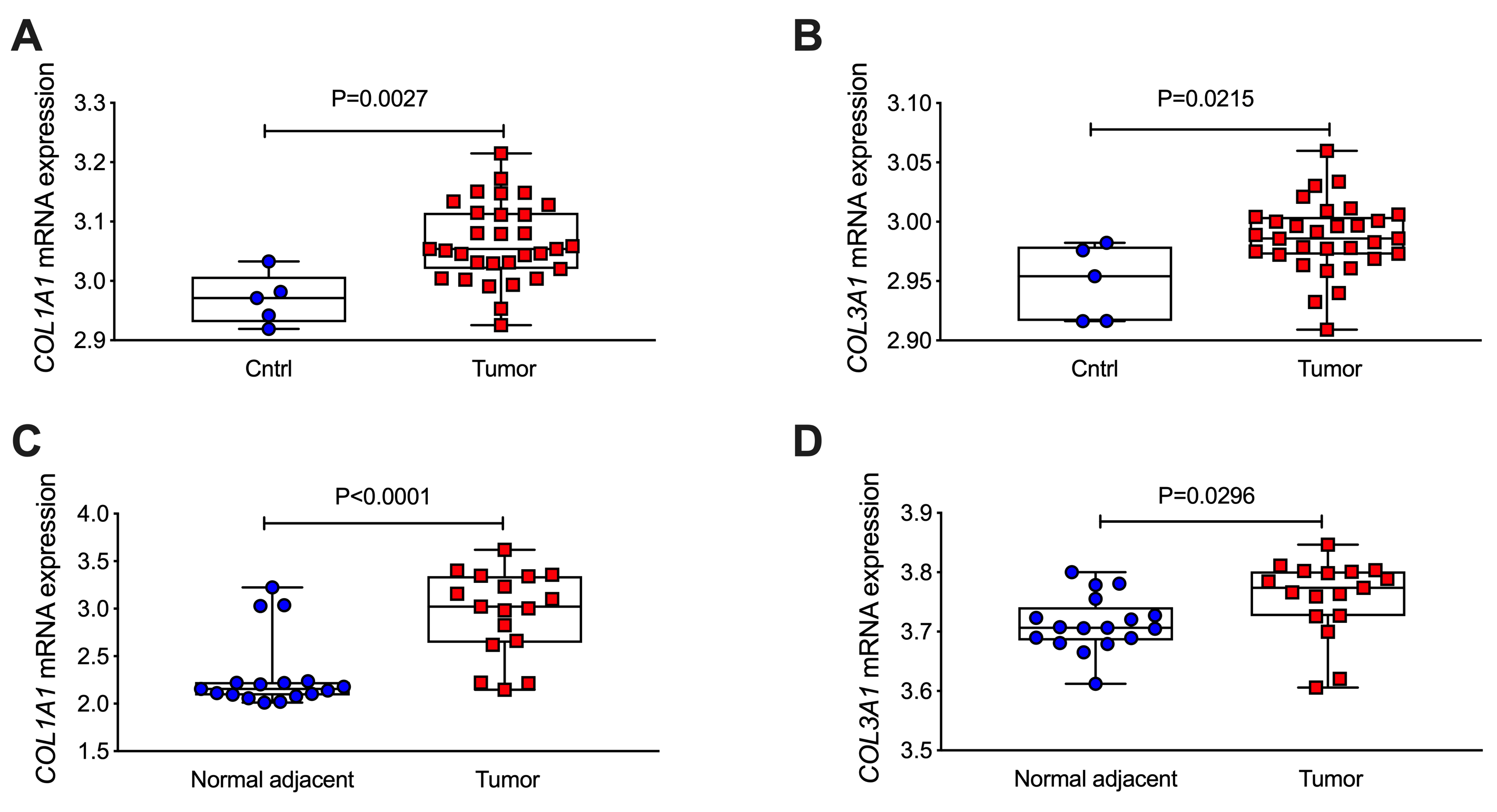


**Fig. S1**. Type I alpha 1 collagen (COL1A1) and type III alpha 1 collagen (COL3A1) mRNA expression are increased in tumor tissues compared to adjacent non-tumor colon tissues from colorectal cancer (CRC) patients. *COL1A1* (A) and *COL3A1* (B) mRNA expression was assessed from colon tissues from healthy controls (n=5) and CRC patients (n=31) based on a GSE128449 dataset. *COL1A1* (C) and *COL3A1* (D) gene expression was measured from colon tissues from tumor and adjacent non-tumor normal tissues from the same CRC patients (n=17) according to a GSE110224 dataset. Results are mean ± SEM.


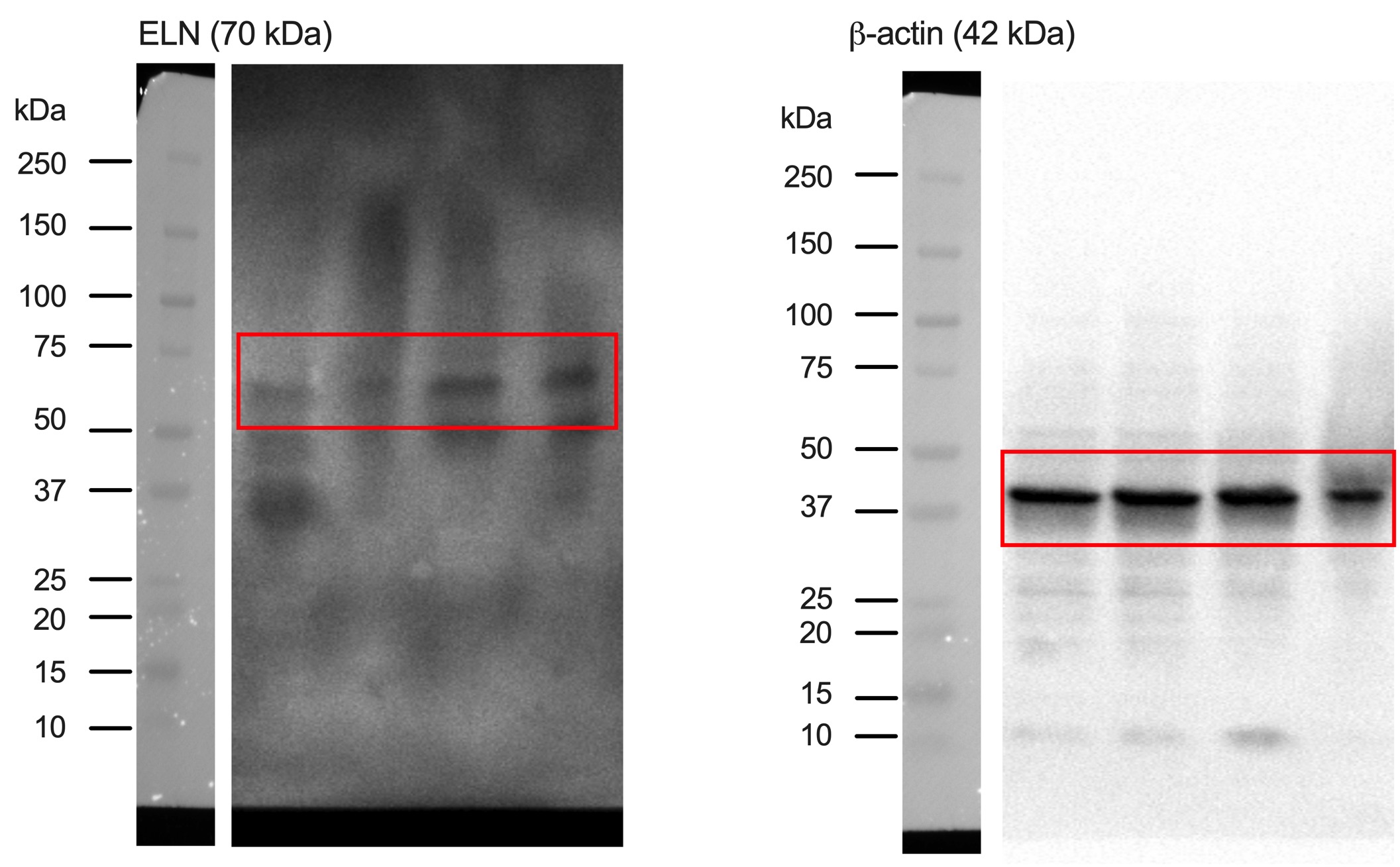


**Fig. S2**. Full length immunoblots of ELN and β-actin in **Fig. 1c**. ELN was probed and the same blot was stripped for β-actin detection by immunoblot Molecular ladder was shown on the side of each immunoblot image. Red rectangle indicates the cropped representative image in **Fig. 1c**.


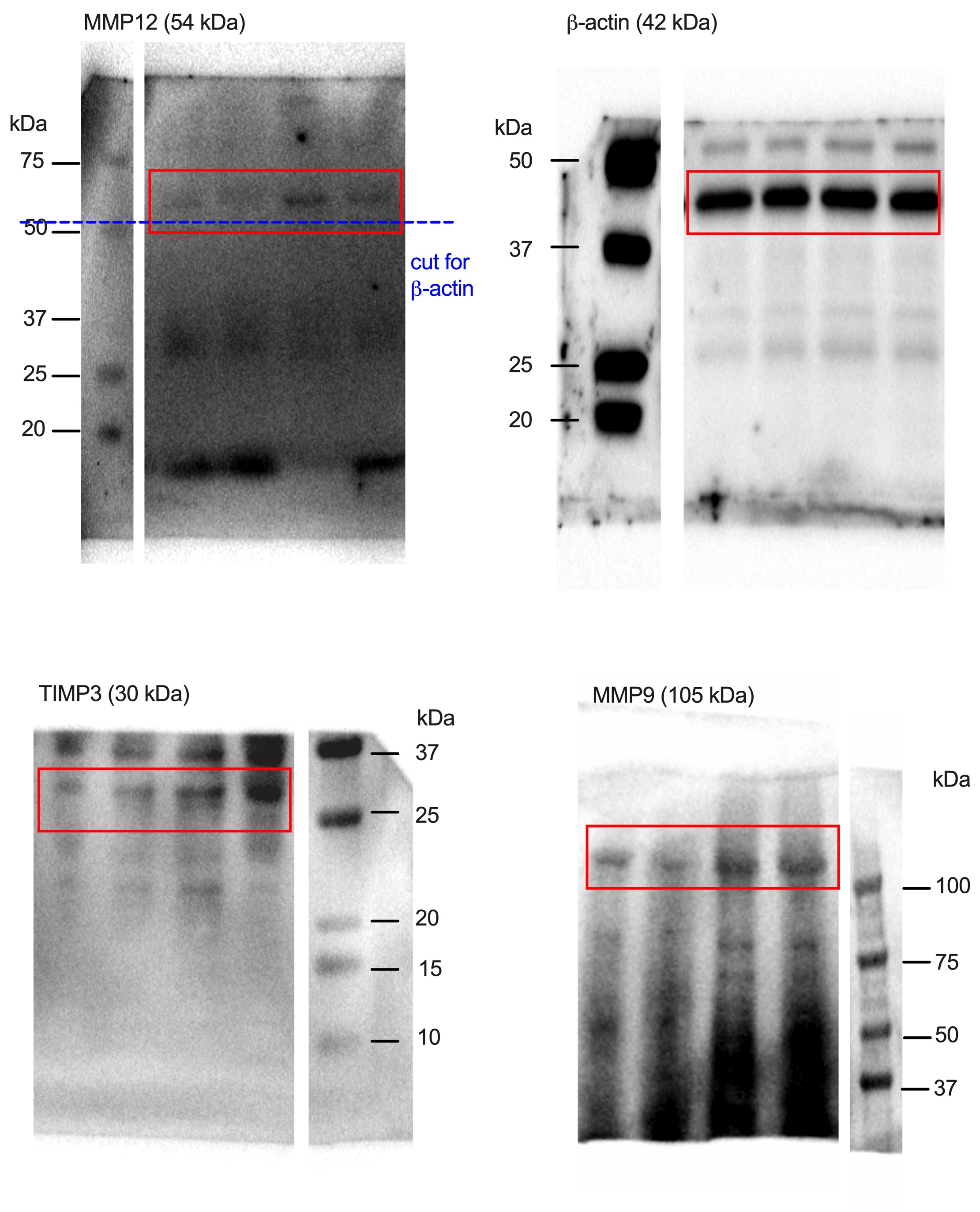


**Fig. S3**. Full length immunoblots of MMP12, MMP9, TIMP3 and β-actin in **Fig. 3**. Molecular ladder was shown on the side of each immunoblot image. Red rectangle indicates the cropped representative image in **Fig. 3**. MMP12 blot were cut and incubated with β-actin after stripping.
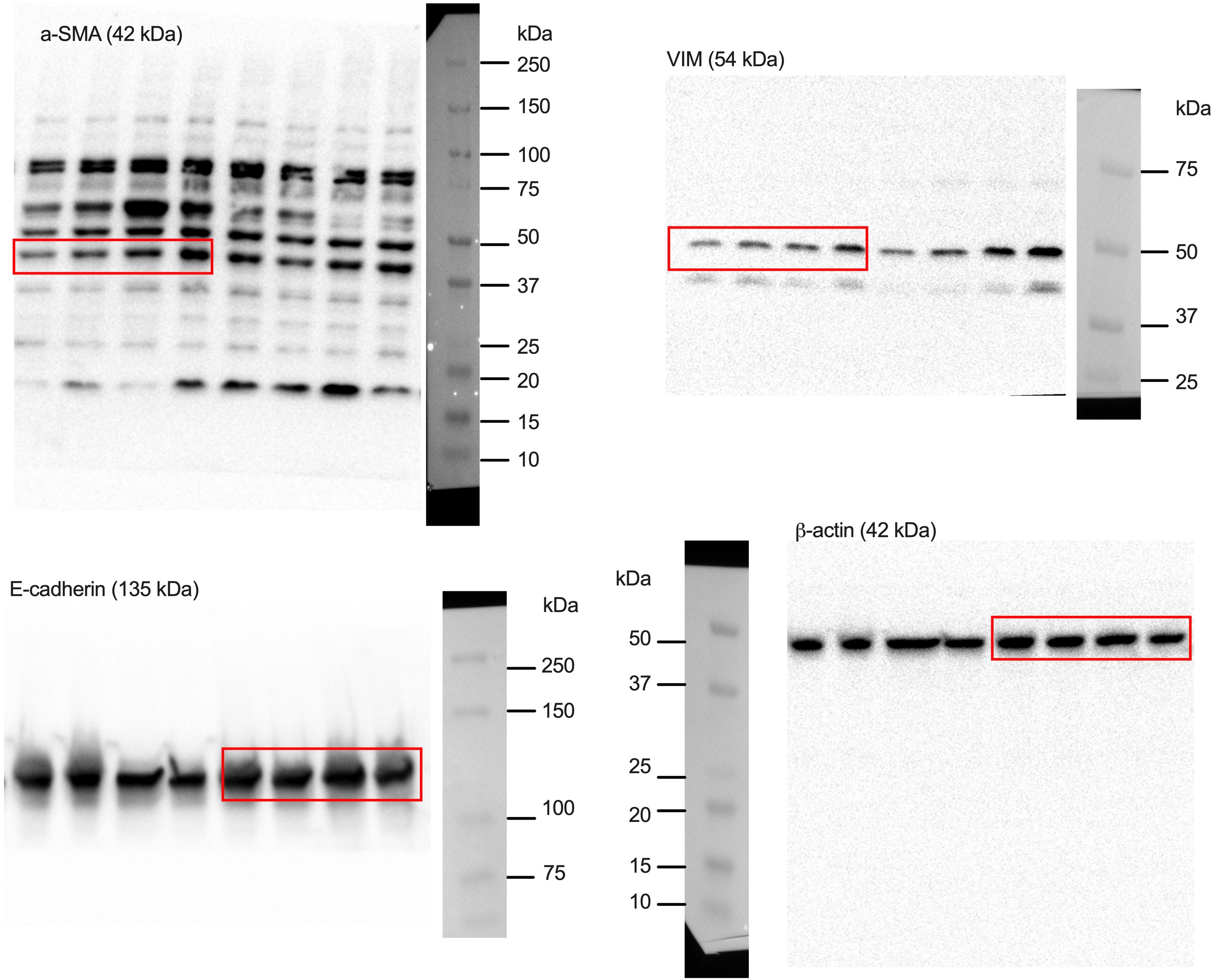


**Fig. S4**. Full length immunoblots of α-SMA, VIM, E-cadherin and β-actin in **Fig. 5**. Molecular ladder was shown on the side of each immunoblot image. Red rectangle indicates the cropped representative image as shown in **Fig. 5**.
